# Myxoglobulosis of the Appendix: A Case Associated with Ruptured Diverticulum

**DOI:** 10.1155/2010/745021

**Published:** 2010-09-01

**Authors:** Panagiotis Aroukatos, Dionysios Verras, Gerassimos P. Vandoros, Maria Repanti

**Affiliations:** Department of Pathology, “Agios Andreas” General Hospital, Tsertidou 1, 26 335 Patras, Greece

## Abstract

We describe a case of the extremely rare entity of myxoglobulosis of the appendix from a 45-year-old white man who was operated urgently with the clinical diagnosis of acute appendicitis. Sectioning of the appendix revealed the presence in the dilated appendiceal lumen of numerous whitish opaque globules ranging in size from 0.2 to 0.7 cm in diameter. A ruptured diverticulum and several smaller ones were also found. On microscopic examination, the globules consisted of faintly eosinophilic laminations of mucin surrounding an amorphous granular core. The mucin was identified by positivity with histochemical mucin stains. After thorough microscopic examination of the appendix, our case was diagnosed as myxoglobulosis due to mucosal hyperplasia, associated with ruptured diverticulum and acellular extra-appendiceal mucin deposits.

## 1. Introduction

Our case is a “mucocele” of the appendix associated with extensive mucosal hyperplasia and ruptured diverticulum [1], with the rare accompanying finding of myxoglobulosis. Mucocele alone does not constitute a rare finding in routine appendectomy. However, the coexistence of multiple small intraluminal globules, described in the past as “frog eggs” or “fish eggs”, constitutes a special type of mucocele described as “myxoglobulosis” [2].

## 2. Case Presentation

**Figure 1:**
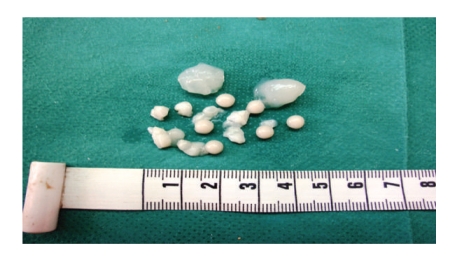
Macroscopic appearance of the extruded mucin and globules.

**Figure 2:**
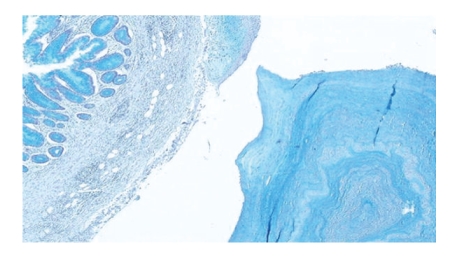
Alcian Blue positive staining of the globules, adjacent luminal mucin and mucosa (Alcian Blue pH 2.5, x100).

A 45-year-old white man entered the hospital on October 19, 2009 and was operated urgently with the clinical diagnosis of acute appendicitis. The pathology specimen consisted of a dilated appendix measuring 6 cm in length and 2 cm in diameter, adherent to the mesoappendix. Sectioning of the appendix revealed the presence in the dilated lumen of numerous whitish opaque globules ranging in size from 0.2 to 0.7 cm in diameter. The mesoappendix near the distal portion of the appendix showed mucin deposits and globules, which appeared to originate from a ruptured diverticulum (Figure 1). Several smaller nonruptured diverticula were also found. The entire appendix was submitted for histological evaluation. Hematoxylin-Eosin and histochemical stains Mucicarmine, Alcian Blue pH 2.5, PAS, and Diastase-PAS were performed. Histologically, the globules consisted of faint eosinophilic laminations of mucin surrounding an amorphous granular core. All of the globules stained uniformly positive with mucicarmine, Alcian Blue and PAS and were diastase resistant (Figure 2). Exuberant hyperplasia of the appendiceal mucosa was also identified. A diagnosis of Low-Grade Appendiceal Mucinous Neoplasm (LAMN), Low Risk of Recurrence [3] was excluded, as mucosal architecture was mainly preserved in all sections examined. Crypts were separated by adequate lamina propria and exhibited mild disarray. Focal gland serration and increased mucin-producing cells were found in the upper half of the mucosa, instead of slender filiform villi that are usually observed in LAMNs. Due to concomitant acute inflammation, focal epithelial crowding of the villi was interpreted as reactive atypia rather than low-grade dysplasia. Around the ruptured diverticulum, mucin had been extruded through the appendiceal wall and mesoappendiceal fat onto the serosa and accompanied by a prominent foreign-body giant cell inflammatory reaction. In serial sections examined microscopically, the periappendiceal mucin did not contain any epithelial elements. Thus the diagnosis of acellular extra-appendiceal mucin was set. As no molecular analysis was performed to exclude with certainty LAMN, a recommendation was given that the patient be followed up to exclude recurrence with mucinous ascites.

## 3. Discussion

Myxoglobulosis of the appendix is characterized by the presence of mucinous, often calcified, pear-like globules in the lumen of the appendix. The first case of mucocele with myxoglobulosis was described by Latham in 1897 in an autopsy case. The incidence of myxoglobulosis is between 0.35% and 8% of all mucoceles [2, 4]. In a study of 50,000 appendectomy specimens, Collins [5] found eight cases of myxoglobulosis, or approximately one in 6,200 removed appendices. In contrast, Hollstrom [4] reported an incidence of only one in 47,500 appendectomies.

Most cases of myxoglobulosis have been incidental findings at autopsy or laparotomy while a few have presented clinically as a “surgical abdomen,” most commonly acute appendicitis. Perforation of the appendix has not been an infrequent complication. However, the most usual complication is generally considered either peritonitis or pseudomyxoma peritonei [4, 6]. The factors leading to the transformation of mucin into globular bodies of myxoglobulosis are unknown. Various hypotheses have focused on the formation of a core, which then acts as a nidus for the concentric deposition of mucin, as the initiating event in the pathogenesis of the globules. Some authors propose the hypothesis of bacterial and necrotic epithelial debris origin and small mucinous masses putatively formed in dilated glandular crypts [2, 4, 6]. Lubin and Berle [7] in a report of two cases proposed that the core represented an organizing mass of mucin and granulation tissue originating in the appendiceal wall that broke off and underwent necrosis. In our case, examination of all the globules failed to reveal any viable cellular elements. The same general mechanisms requisite in the production of appendiceal mucocele are involved in the pathogenesis of myxoglobulosis. These mechanisms include (a) partial or complete obstruction of the lumen of the appendix and (b) continued mucin production by a normal or altered—for example, neoplastic—epithelium.

 Therefore mucoceles of the appendix related to retention cysts, focal or diffuse mucosal hyperplasia, mucinous cystadenoma, and mucinous cystadenocarcinoma may present with myxoglobulosis. The majority of the cases of myxoglobulosis belongs to the category of mucosal hyperplasias and the retention cyst type of mucocele [6]. Viswanath et al. [8] reported a case of myxoglobulosis secondary to an occlusive membrane while Brustmann [9] described another one associated with a proximal carcinoid. Lo and Kan [10] described a case of appendiceal mucinous cystadenoma presented as “porcelain” appendix with myxoglobulosis, and Guionmet et al. [11] reported a case of mucinous cystadenoma of the appendix with myxoglobulosis associated to cecal adenocarcinoma. 

 After thorough microscopic examination of the appendix, our case was diagnosed as myxoglobulosis due to mucosal hyperplasia, associated with ruptured diverticulum and acellular extra-appendiceal mucin deposits.
